# From HITS to misses: aspirin's effect on TCD-detected cerebral microemboli in Chagas cardiomyopathy

**DOI:** 10.1055/s-0045-1812885

**Published:** 2025-12-02

**Authors:** Brandon J. Bond, Victoria Grau Kazmieczak, José Biller

**Affiliations:** 1 Loyola University Health System, Loyola University Chicago, Stritch School of Medicine, Department of Neurology, Maywood, Illinois, United States; 2 Loyola University Health System, Loyola University Chicago, Stritch School of Medicine, Department of Neurological Surgery, Maywood, Illinois, United States

Chagas disease, caused by the protozoan *Trypanosoma cruzi* , remains a major public health challenge in Latin America and is increasingly recognized in non-endemic regions due to migration. 1 Affecting over 7 million people globally, it is a leading cause of non-ischemic cardiomyopathy and heart failure (HF) in endemic countries, with up to one-third of infected individuals developing cardiac involvement. 1 2

Chagas cardiomyopathy carries a substantial risk of arrhythmias, sudden death, and thromboembolic events, particularly stroke. 1 3 Epidemiologic studies identify Chagas disease as an independent predictor of ischemic stroke—HR 2.54 (95% CI 1.01–6.42) in a heart-failure cohort, and OR 7.17 (95% CI 1.50–34.19) in a case–control study. 3 4 Proposed mechanisms include chronic inflammation, endothelial dysfunction, and a prothrombotic state contributing to intracardiac thrombus—particularly in patients with a left ventricular apical aneurysm or reduced left ventricular ejection fraction (LVEF). 1

Transcranial Doppler (TCD) ultrasonography enables the detection of cerebral microembolic signals—high-intensity transient signals (HITS), also termed microembolic signals (MES)—which are considered markers of subclinical embolic phenomena traversing the cerebral circulation. 5 These signals have been correlated with elevated early stroke risk in patients with carotid stenosis and occlusion. 6 TCD-detected microembolic signals are more frequent in Chagas cardiomyopathy than in other causes of HF (12.9% vs 1.2%) and this association is independent of LVEF, suggesting additional mechanisms—including chronic inflammation—may contribute to cerebral embolic activity. 7 Yet, whether this microembolic activity is modifiable through antithrombotic therapy in Chagas cardiomyopathy has remained unclear.

In this context, the pilot randomized controlled trial by Castello-Branco and colleagues offers compelling preliminary evidence that acetylsalicylic acid (ASA, or aspirin) may mitigate cerebral microembolism in Chagas HF. 8 Conducted using a prospective, randomized, open-label, blinded-endpoint (PROBE) design, the study enrolled patients with Chagas HF and HITS on baseline TCD, randomizing them 2:1 to receive ASA 300 mg daily for seven days plus standard therapy or standard therapy alone. The cohort reflected a relatively low thromboembolic risk profile: all were in sinus rhythm (atrial fibrillation [AF] excluded), had no prior stroke, and were not anticoagulated at baseline, though some were taking antiplatelet agents. The primary endpoint was the persistence of HITS after one week, assessed by a blind investigator.

Of 373 HF patients screened (190 with Chagas disease), HITS were identified in 11%, without significant difference between Chagas (12%) and non-Chagas (8%) groups after multivariable adjustment for age, sex, and LVEF (odds ratio 1.33, 95% CI 0.60–2.95). Among the 22 Chagas patients with HITS, 12 were randomized (8 to ASA, 4 to control). After 7 days, none of the ASA-treated patients exhibited persistent HITS, whereas two of four controls (50%) continued to do so ( *p* = 0.028). Median HITS decreased from 3.5 to 0 in the ASA group ( *p* = 0.012) but showed a nonsignificant trend in controls (4.0 to 0.5, *p* = 0.095). This suggests a possible short-term biological effect of aspirin, though the difference between groups did not reach statistical significance ( *p* = 0.262).

These findings are consistent with previous work demonstrating aspirin's capacity to reduce cerebral microemboli in other embolic disorders. Goertler et al. showed that intravenous ASA (500 mg) significantly reduced MES within 30 minutes in 9 patients with recent TIA or minor stroke of arterial origin. 9 Low-dose aspirin irreversibly acetylates platelet COX-1 and suppresses thromboxane A _2_ , and doses of 300–500 mg produce near-complete inhibition of platelet aggregation, plausibly explaining the rapid MES reduction observed. 10 11 Beyond platelet inhibition, experimental data point to anti-inflammatory and endothelial benefits of aspirin in *T. cruzi* infection. In murine Chagas models, ASA improves endothelial function, reduces cardiac inflammation, and lowers thromboxane levels, possibly through 15-epi-lipoxin A _4_ –mediated anti-inflammatory signaling. 12 13 When combined with benznidazole, an antiparasitic drug used to treat Chagas disease, ASA prevents cardiovascular dysfunction and decreases chronic cardiac fibrosis. 14

Despite its promise, this trial's limitations merit careful consideration. The randomized sample was very small ( *n* = 12), below the prespecified target of 30 due to logistical barriers and early termination. Its 7-day follow-up precludes conclusions about sustained benefit or stroke reduction. Moreover, the study did not replicate prior reports 7 of higher HITS prevalence in Chagas versus non-Chagas HF (12% vs 8%, *p* = 0.531, adjusted OR 1.33 [95% CI 0.60–2.95]), a result that likely reflects a lower-risk cohort with many patients in sinus rhythm, exclusion of prior stroke, and comparatively preserved LVEF in the randomized subset. Additionally, female predominance and milder HF severity may also have attenuated embolic risk.

Another consideration is safety. Although no adverse events were observed, prior analyses show that aspirin can increase bleeding in advanced heart failure, and in LVAD-supported patients, avoiding aspirin did not raise thromboembolic events while reducing major bleeding. 15 16 Broader reviews also detail aspirin's bleeding toxicity and discuss possible prostaglandin-mediated hemodynamic effects. 15 16 Nevertheless, meta-analyses show that aspirin use is associated with a small absolute increase in major bleeding—∼0.47% over 5 years in primary prevention. 17 Brazilian guidelines recommend oral anticoagulation in Chagas cardiomyopathy for AF (per CHA _2_ DS _2_ -VASc), documented LV mural thrombus, or prior ischemic stroke. Apical LV aneurysm is a risk marker incorporated into the IPEC-FIOCRUZ score but is not, by itself, anticoagulation indication, and routine antiplatelet therapy in sinus rhythm is not recommended for cardioembolic prevention. 18 Consistent with contemporary AHA/ASA guidance, randomized trials in heart failure with reduced LVEF and no AF show no clear net benefit of routine anticoagulation—stroke reductions are modest while bleeding increases—and there are no data that antiplatelet therapy prevents first stroke in this setting. 19

From a translational perspective, using TCD as a surrogate endpoint is valuable for proof-of-concept trials, offering a non-invasive, real-time biomarker of cerebral embolic activity. This kind of monitoring can inform study design and future clinical testing of emboli-targeted strategies. 20

In conclusion, Castello-Branco et al. takes an important first step toward linking vascular biology with clinical translation in Chagas heart failure. Although the sample is small and follow-up is brief, their pilot randomized data suggest that aspirin 300 mg daily may reduce cerebral microemboli—an early, potentially modifiable marker of cerebrovascular risk. Larger multicenter studies with longer follow-up and clinical outcomes such as stroke and mortality are essential to confirm these observations. In the broader fight against neglected tropical diseases, even modest interventions, when rigorously tested, can illuminate new paths for global stroke prevention.
